# Gray Matter Alterations in Parkinson's Disease With Rapid Eye Movement Sleep Behavior Disorder: A Meta-Analysis of Voxel-Based Morphometry Studies

**DOI:** 10.3389/fnagi.2020.00213

**Published:** 2020-08-12

**Authors:** Chengxian Yang, Jianbo Chang, Xiaohang Liang, Xinjie Bao, Renzhi Wang

**Affiliations:** ^1^Department of Neurosurgery, Peking Union Medical College Hospital, Chinese Academy of Medical Sciences and Peking Union Medical College, Beijing, China; ^2^Center for MRI Research, Academy for Advanced Interdisciplinary Studies, Peking University, Beijing, China

**Keywords:** voxel-based morphometry, gray matter, neuroimaging, sleep disorder, Parkinson's disease

## Abstract

**Background:** Gray matter (GM) alterations in Parkinson's disease (PD) patients with rapid eye movement sleep behavior disorder (RBD) have been demonstrated in many neuroimaging studies using voxel-based morphometry (VBM). However, the inconsistent findings between studies cannot be applied to clinical practice as a neuroimaging biomarker. We performed a meta-analysis of VBM studies at a whole-brain level to investigate GM differences between PD patients with and without RBD.

**Methods:** A systematic search was conducted in PubMed, Embase, and Web of Science from inception to November 2019 to identify eligible VBM studies. We adopted the latest Seed-based *d* Mapping with Permutation of Subject Images technique to quantitatively estimate the difference of regional GM volume between PD patients with and without RBD.

**Results:** We included five studies comprising 105 PD patients with RBD and 140 PD patients without RBD. The pooled meta-analysis revealed that PD patients with RBD showed a significant reduction of GM volume in the right superior temporal gyrus (STG) compared with those without RBD. This result was confirmed to be robust by the jackknife sensitivity analysis.

**Conclusion:** Our finding shows significantly and robustly reduced GM volume in the right STG in PD patients with RBD, preliminarily suggesting the association of GM atrophy in this brain region with the occurrence of RBD in PD patients.

## Introduction

Parkinson's disease (PD) is the second most common neurodegenerative disorder with a rising prevalence in parallel with an aging population (Zhang et al., 2005; Pringsheim et al., 2014). Though the clinical diagnosis of PD is mainly based on cardinal motor symptoms including bradykinesia, rigidity, and rest tremor (Postuma et al., 2015), non-motor symptoms, such as hyposmia, depression, sleep disorders, and constipation, have attracted increasing attention because of their negative influence on the quality of life and predictive value for disease progression (Schapira et al., 2017). The treatment of PD remains a massive challenge because there has been no reliable tool for the early detection of PD worsening. However, the non-motor symptoms of PD can occur several years before motor manifestation progression; therefore, monitoring non-motor symptoms is considered a promising method to evaluate disease risks and promote the clinical intervention of PD at an early stage.

Rapid eye movement sleep behavior disorder (RBD) is a parasomnia characterized by loss of skeletal muscle atonia and dream-enacting behaviors associated with aggression and violence during rapid eye movement sleep (St Louis and Boeve, 2017). A handful of studies have demonstrated that RBD is strongly associated with PD. The prevalence of RBD is about 30% to 50% in patients with PD (Howell and Schenck, 2015). The occurrence of RBD in PD is associated with motor function deterioration (particularly bradykinesia worsening) (Bugalho and Viana-Baptista, 2013), more severe non-motor symptoms (including anxiety, depression, sleep disorders, constipation, hallucination, and orthostatic hypotension) (Neikrug et al., 2014; Liu et al., 2017), poorer cognitive function (Huang et al., 2017; Jozwiak et al., 2017), and cerebral cortex abnormalities (Barber et al., 2017). Thus, the presence of RBD symptoms is a risk factor and a potential marker of disease progression in PD.

Previously, many magnetic resonance imaging (MRI) studies have been conducted to investigate the functional and structural brain alterations in PD with RBD in the hope of uncovering the potential pathophysiology and characteristic changes in the brain (Bourgouin et al., 2019). However, due to the limited sample size and different analytical methods, the mixed results of these studies cannot be a neuroimaging biomarker in clinical practice.

Seed-based *d* mapping (SDM) is a fully validated coordinate-based meta-analytic method, which can synthesize data of neuroimaging studies using functional MRI, voxel-based morphometry (VBM), diffusion tensor imaging, or positron emission tomography (Albajes-Eizagirre et al., 2019b). In the present study, we focused on regional volume changes of gray matter (GM) using VBM analysis. The current study aimed to calculate structural alterations in PD with RBD from published data to identify a robust neuroimaging biomarker. Therefore, we performed a coordinate-based meta-analysis of VBM studies at a whole-brain level to elucidate the prominent GM changes in PD patients with RBD.

## Materials and Methods

The present systematic review and meta-analysis was performed according to the Preferred Reporting Items for Systematic Reviews and Meta-Analysis (PRISMA) guidelines. We prepared a prospective protocol of search strategy, inclusion criteria, data extraction, and methods of statistical analysis.

### Literature Search

We performed a systematic and comprehensive search of the PubMed, Embase, and Web of Science from inception to November 3, 2019. For the literature search in these databases, the following terms were used in combinations: (“magnetic resonance imaging” or “MRI”) and (“Parkinson disease” or “Parkinson's disease”) and (“rapid eye movement sleep behavior disorder” or “REM sleep behavior disorder” or “RBD”). Additionally, we checked the references of relevant original articles and reviews. The language of publications was restricted to English. To avoid time-lag bias, the search process was updated in June 12, 2020, but no additional study was identified.

### Inclusion Criteria

We included studies in the meta-analysis that met the following criteria: (1) was published as an original article in a peer-reviewed journal; (2) demonstrated specific diagnostic criteria of PD and RBD; (3) conducted a whole-brain analysis of GM structural alterations in a stereotactic space in three-dimensional standard coordinates; (4) reported the results of VBM analysis in PD patients with and without RBD; and (5) reported significance thresholds that were either corrected for multiple comparisons or uncorrected with spatial extent thresholds. Therefore, editorials, letters, conference abstracts, reviews, book chapters, and case reports were excluded. Two authors (C.Y. and J.C.) reviewed the studies independently.

### Data Extraction

We extracted peak coordinates of abnormal brain regions from all studies eligible for meta-analysis. If the manuscript used two whole-brain statistical significance levels with and without correction for multiple comparisons, we selected the uncorrected threshold and included all peaks obtained using this uncorrected threshold (Albajes-Eizagirre et al., 2019a,b). The following data were also collected: the number of patients in each group, mean age, year of education, gender ratio, disease duration, severity, and diagnostic criteria of RBD and PD. All data were evaluated independently by two authors (C.Y. and J.C.). Any disagreement about literature search, study selection, and data extraction was resolved by consensus under the guidance of the senior authors (X.B. and R.W.).

### Quality Assessment

Each study was assessed for quality and completeness using a 13-item checklist as described in previous meta-analyses (Baiano et al., 2007; Shepherd et al., 2012; Du et al., 2014). The 13 items were divided into three categories, namely, subjects, methods for image acquisition and analysis, and results and conclusions. Each item was rated 1, 0.5, or 0 if criteria were fully met, partially met, or unfulfilled, respectively (Baiano et al., 2007). The checklist was designed to rate the completeness of neuroimaging studies, providing an objective indication of rigor of included studies in a meta-analysis.

### Data Analysis

We conducted the voxel-wise meta-analysis of regional differences in GM volume between PD patients with and without RBD using SDM with Permutation of Subject Images (SDM-PSI version 6.21) (Albajes-Eizagirre et al., 2019a,b). The detailed coordinate-based meta-analytic process of VBM results has been described in the software tutorial (https://www.sdmproject.com/) and previous studies (Albajes-Eizagirre et al., 2019a; Dahlgren et al., 2020). Here, we briefly summarized the SDM-PSI methods. First, the peak coordinates and *t*-statistics of GM differences between PD patients with and without RBD were extracted. To avoid potential bias, we ensured that the same statistical threshold was used in the whole brain in each study. If *t*-statistics were not presented in the publications, *z*-scores or *p*-values were converted into *t*-statistics by the SDM-PSI software. Second, the lower and upper bounds of potential effect sizes for all voxels were estimated by multiple imputations, and maps of GM alterations for each study were created by means of an anisotropic Gaussian kernel, which assigns higher effect sizes to the voxels more correlated with peak coordinates. Third, maximum likelihood techniques were used to estimate the most likely effect size and its standard error. The imputed dataset from each study was meta-analyzed using a random-effects model, and these imputed meta-analyzed datasets were combined using Rubin's rules. Fourth, we conducted family-wise error correction for multiple comparisons and thresholded the meta-analysis using threshold-free cluster enhancement statistics. The following SDM-PSI parameters were used: full width at half maximum (FWHM) = 20 mm, voxel size = 2 mm, imputations = 50, permutations = 1,000, threshold-free cluster enhancement family-wise error rate *P* = 0.05, cluster extent threshold = 10 voxels. Egger's tests and funnel plots were used to evaluate publication bias, and heterogeneity of effect sizes was assessed by *I*^2^ statistics. Besides, we performed jackknife sensitivity analyses, in which the meta-analysis was repeated after discarding one eligible study at a time to see whether the result remained statistically significant.

## Results

**Figure 1:**
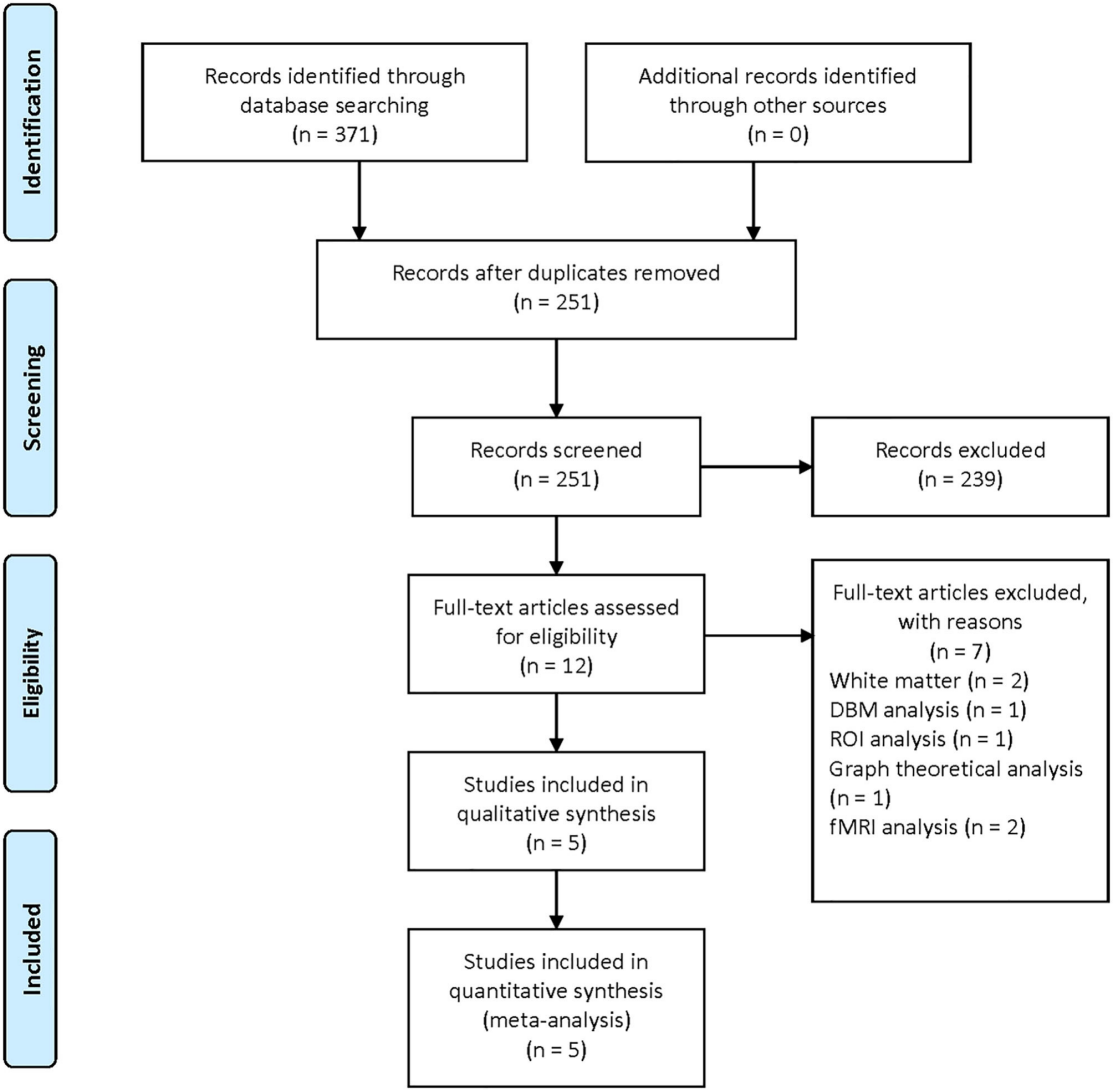
Flow diagram illustrating the results of the study selection and inclusion in the current meta-analysis.

The literature search yielded 371 publications (PubMed: 71; Embase: 200; Web of Science: 100). After screening titles and abstracts, 12 studies were reviewed in full texts. In the end, five studies were included in the current meta-analysis (Ford et al., 2013; Salsone et al., 2014; Kim et al., 2016; Lim et al., 2016; Rahayel et al., 2019). The process of study selection is presented in Figure 1.

### Characteristics of Included Studies

There are a total of 105 PD patients with RBD and 140 without RBD. The baseline data about age, gender, education, disease duration, and disease severity were comparable between groups in each study. The diagnosis tool of PD is UK Brain Bank in four studies from 2013 to 2016 and Movement Disorder Society clinical criteria in one study in 2019. The clinical data and quality assessment scores of five included studies are presented in Table 1. MRI parameters are demonstrated in Table 2. Statistical parameters and results of VBM analyses are shown in Table 3. The details of quality assessment in terms of subjects, methods for image acquisitions and analysis, and results and conclusions are shown in Table S1.

**Table 1:** Demographic and clinical data of five included studies in the present meta-analysis.

**References**	**Country**	**No. of patients**		**Age, years**		**Duration of illness,** **years**		**Education, years**		**Gender, men (%)**		**Severity, Hoehn and** **Yahr stage**		**Levodopa equivalent** **dose, mg**		**UPDRS part 3** **score**		**Diagnosis of PD**	**Diagnosis of RBD**	**Quality Score**
		**PDRBD+**	**PDRBD–**	**PDRBD+**	**PDRBD–**	**PDRBD+**	**PDRBD–**	**PDRBD+**	**PDRBD–**	**PDRBD+**	**PDRBD–**	**PDRBD+**	**PDRBD–**	**PDRBD+**	**PDRBD–**	**PDRBD+**	**PDRBD–**			
Ford et al. (2013)	UK	46	78	66.4 ± 9.9	65.8 ± 10.9	6.5 ± 5.1	6.0 ± 4.4	13.0 ± 3.6	13.0 ± 4.1	36 (78.3)	48 (61.5)	2.15 ± 0.73	1.91 ± 0.56	179.2 ± 144.7	172.6 ± 128.2	26.3 ± 10.0	27.3 ± 11.9	UK Brain Bank	Mayo Sleep Questionnaire	11.5
Salsone et al. (2014)	Italy	11	11	66.6 ± 7.4	66.9 ± 7.9	4.72 ± 4.07	4.36 ± 4.2	NA	NA	8 (72.7)	8 (72.7)	1.95 ± 0.57	1.86 ± 0.59	563.6 ± 167.3	547.7 ± 155.4	21.6 ± 10.4	19.9 ± 10.3	UK Brain Bank	PSG	13
Kim et al. (2016)	Korea	9	22	70.1 ± 6.8	67.7 ± 8.4	1.9 ± 1.5	1.9 ± 1.4	2.4 ± 2.5	8.5 ± 4.8	0 (0)	10 (45.5)	≤ 3	≤ 3	NA	NA	NA	NA	UK Brain Bank	PSG	13
Lim et al. (2016)	Korea	24	14	69.8 ± 6.4	69.7 ± 7.2	6.2 ± 2.9	4.4 ± 3.7	NA	NA	12 (50)	8 (57.1)	1.9 ± 0.4	1.6 ± 0.5	NA	NA	12.4 ± 2.5	22.4 ± 10.6	UK Brain Bank	PSG	12
Rahayel et al. (2019)	Canada	15	15	66.7 ± 7.6	63.1 ± 8.9	3.9 ± 2.9	3.7 ± 2.6	14.2 ± 3.6	15.7 ± 3.9	10 (66.7)	5 (33.3)	NA	NA	625.2 ± 347.1	447.8 ± 171.5	24.1 ± 10.0	17.6 ± 8.5	MDS criteria	PSG	13

*PD, Parkinson's disease; RBD, rapid eye movement sleep behavior disorder; MDS, Movement Disorder Society; PSG, polysomnography. Data are presented with mean ± standard deviation*.

**Table 2:** Magnetic resonance imaging parameters of five included studies in the present meta-analysis.

**Study**	**Manufacturer**	**Model**	**Field strength (T)**	**Head coil**	**FA (**°**)**	**TI (ms)**	**TE (ms)**	**TR (ms)**	**Sequence**	**RR (mm)**
Ford et al.	Philip	NA	3	NA	NA	NA	NA	NA	MPRAGE	1.15 × 1.15 × 1.2
Salsone et al.	GE	MR-750	3	8-channel head coil	12	NA	9.2	3.7	Spoiled gradient echo	1 × 1 × 1
Kim et al.	Phillip	Achieva	3	Standard quadrature head coil	13	NA	2.7	7.3	Spoiled fast gradient echo	Slice thickness 1
Lim et al.	Philip	Achieva	3	NA	8	NA	4.6	9.9	NA	Slice thickness 1
Rahayel et al.	Siemens	TrioTIM	3	12-channel head matrix coil	9	900	2.91	2300	MPRAGE	1 × 1 × 1

*FA, flip angle; TI, inversion time; TE, echo time; BW, bandwidth per pixel; TR, repetition time; MPRAGE, magnetization prepared rapid gradient echo; RR, reconstructed resolution*.

**Table 3:** Statistical parameters and results of VBM analyses in five included studies.

**Study**	**Region description**	**MNI Coordinates**			**Voxel**	**Covariate**	**Threshold**
		***x***	***y***	***z***			
Ford et al.	Left parietal operculum	−62	−7	9	407	Age, gender, intracranial volume	*P* < 0.001, uncorrected
	Right superior temporal gyrus	52	−28	13	191		
	Left insula cortex	−32	−15	12	146		
	Right hippocampus	36	−10	−14	138		
	Left middle occipital gyrus	−39	−91	0	131		
Salsone et al.	Right thalamus	−9	−21	15	85	Age, intracranial volume	*P* < 0.001, uncorrected
	Right medial temporal cortex	68	−22	−6	176		
Kim et al.	Right superior temporal cortex	62	−8	8	114	Age, gender, intracranial volume	*P* < 0.001, uncorrected
Lim et al.	Left lingual gyrus	−5	−92	0	90	Age, gender, PD duration, HandY stages	*P* < 0.001, uncorrected
	Right cuneus	12	−68	6	108		
	Left hippocampus	−38	−33	−5	50		
	Left cingulate gyrus	−8	−24	39	303		
Rahayel et al.	Left lingual gyrus/cerebellum	−14	−56	−9	156	Age, gender, education, MCI status, UPDRS-III score	*P* < 0.0036, 14 comparisons

*MNI, Montreal Neurological Institute; PD, Parkinson's disease; MCI, mild cognitive impairment*.

### Outcomes of Data Analysis

**Figure 2:**
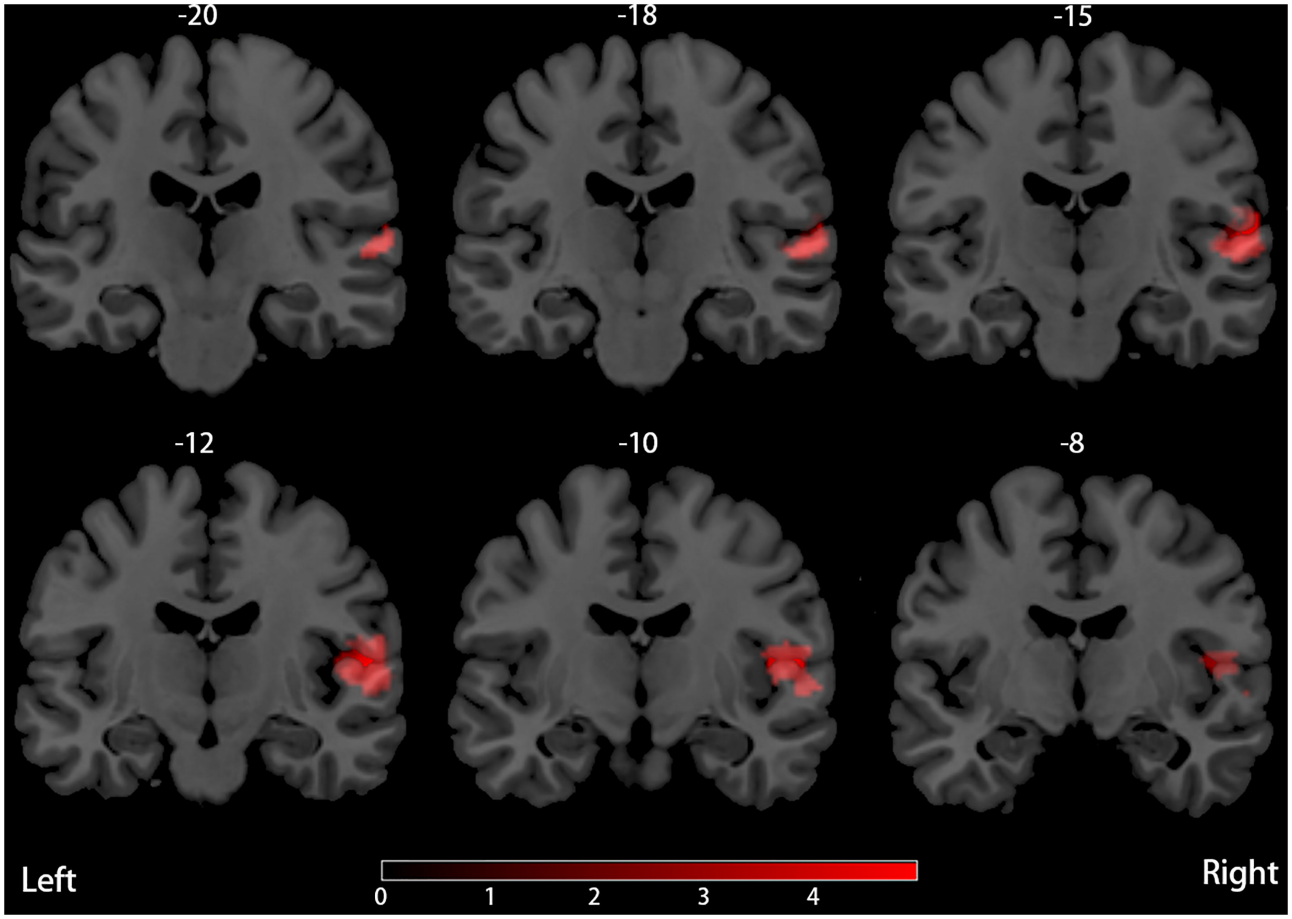
Decreased (red) GM volume in PD patients with RBD compared with those without RBD.

**Figure 3:**
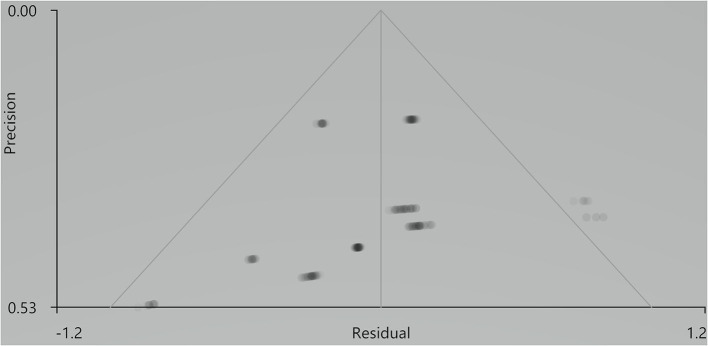
Results of funnel plot analysis to test for publication bias.

Our result demonstrated a significant GM volume reduction in the right superior temporal gyrus (STG) in Figure 2. The cluster breakdown results show which cortical areas contribute to the corresponding cluster. We included the cortical areas with more than 10% voxels of the present cluster using 20 mm FWHM in Table 4. Results of Egger's tests (bias = −0.70, *t* = −0.39, *df* = 3, *P* = 0.699) and funnel plots in Figure 3 revealed no significant publication bias. There was no obvious heterogeneity of effect sizes (*I*^2^ = 2.14%) in the present meta-analysis. The main findings remained largely unchanged in the jackknife sensitivity analysis (3/5), as shown in Table 5.

**Table 4:** Region of smaller GM volume in PD patients with RBD compared with those without RBD.

**FWHM (mm)**	**Local peaks**							**Cluster breakdown**	
	**Region description**	**MNI coordinates (*****x***, ***y***, ***z*****)**			**SDM (*z* Score)**	***P***	**Voxels (*n*)**	**Region description**	**Voxels (*n*, %)**
20	Corpus callosum	58	−16	4	−4.924	~0	366	Right superior temporal gyrus, BA 22	94 (25.7%)
	Right superior temporal gyrus, BA 48	56	−12	8	−4.884	0.000999987		Right rolandic operculum, BA 48	69 (18.9%)
	Right rolandic operculum, BA 48	50	−10	10	−4.353	0.001999974		Corpus callosum	65 (17.8%)
	Right rolandic operculum, BA 22	62	−14	14	−4.311	0.001999974		Right Heschl gyrus, BA 48	41 (11.2%)
	Right superior temporal gyrus, BA 21	64	−32	4	−3.748	0.017000020		Right superior temporal gyrus, BA 48	38 (10.4%)
	Right superior temporal gyrus, BA 21	60	−32	2	−3.714	0.017000020			
	Right superior temporal gyrus, BA 22	58	−26	2	−3.652	0.008000016			

*FWHM, full width at half maximum; BA, Brodmann area; MNI, Montreal Neurological Institute; SDM, seed-based d mapping*.

**Table 5:** Results of jackknife sensitivity analyses.

**Excluded study**	**Region description**	**MNI coordinates**			**SDM (*z* Score)**	***P***	**Voxels (*n*)**	**Cluster breakdown (Voxels, *n*, %)**
		***x***	***y***	***z***				
Ford et al.	NA	NA	NA	NA	NA	NA	NA	NA
Salsone et al.	Right rolandic operculum	56	−12	10	−4.348	0.014999986	61	Right rolandic operculum, BA 48 (16, 26.2%)
	Right rolandic operculum, BA 22	62	−14	14	−4.235	0.023000002		Right Heschl gyrus, BA 48 (12, 19.7%)
	Right Heschl gyrus, BA 48	52	−12	6	−4.221	0.023000002		Right superior temporal gyrus, BA 22 (10, 16.4%)
	Corpus callosum	58	−16	4	−4.057	0.033999979		Right superior temporal gyrus, BA 48 (9, 14.8%)
	Right superior temporal gyrus	64	−16	10	−3.903	0.044000030		
Kim et al.	NA	NA	NA	NA	NA	NA	NA	NA
Lim et al.	Right superior temporal gyrus	58	−14	8	−5.457	0.000999987	297	Right superior temporal gyrus, BA 22 (73, 24.6%)
	Right rolandic operculum, BA 48	56	−12	12	−5.325	0.000999987		Right rolandic operculum, BA 48 (63, 21.2%)
	Right rolandic operculum, BA 22	62	−14	14	−4.608	0.004000008		Corpus callosum (51, 17.2%)
	Right superior temporal gyrus, BA 21	64	−32	4	−3.955	0.024999976		Right superior temporal gyrus, BA 48 (35, 11.8%)
								Right Heschl gyrus, BA 48 (34, 11.4%)
Rahayel et al.	Right superior temporal gyrus, BA 48	58	−16	8	−5.710	0.000999987	335	Right superior temporal gyrus, BA 22 (81, 24.2%)
	Right rolandic operculum, BA 48	56	−12	12	−5.490	0.000999987		Right rolandic operculum, BA 48 (71, 21.2%)
	Right rolandic operculum, BA 22	62	−14	14	−4.834	0.000999987		Corpus callosum (57, 17.0%)
	Right Heschl gyrus, BA 48	48	−12	8	−4.710	0.001999974		Right Heschl gyrus, BA 48 (40, 11.9%)
	Right superior temporal gyrus, BA 21	64	−32	4	−4.066	0.018999994		Right superior temporal gyrus, BA 48 (37, 11.0%)

*NA, not available; MNI, Montreal Neurological Institute; SDM, seed-based d mapping; BA, Brodmann area*.

## Discussion

Up to now, there has been no treatment method to slow or halt the neurodegenerative process in PD. The occurrence of RBD is a warning signal of worsening symptoms in PD patients (Romenets et al., 2012; Mollenhauer et al., 2016). The period between the onset of RBD symptoms and subtle cerebral structural changes is an ideal time window for prompt therapeutic intervention. Herein, we aimed to understand GM changes associated with RBD in PD using VBM.

To the best of our knowledge, this is the first neuroimaging meta-analysis to assess the difference of regional GM volume between PD patients with and without RBD based on VBM studies. The findings of our research can be summarized as follows: there is a significant reduction of GM volume in the right STG in PD patients with RBD compared with those without RBD.

As a newly emerging biomarker, neuroimaging has been extensively investigated for the early diagnosis and prognosis assessment in neurodegenerative disorders (Shimizu et al., 2018). According to human and animal studies, structural lesions in the dorsal midbrain and pons are confirmed to be responsible for idiopathic RBD (Boeve et al., 2007). However, the neuroanatomical basis of RBD in PD is considered different from idiopathic RBD (Dauvilliers et al., 2018). Thus, many studies have been performed to detect specific imaging biomarkers in PD patients before the presence of RBD using multimodal brain MRI methods (Lim et al., 2016; Ansari et al., 2017; Li et al., 2017), among which VBM using high-solution T1 imaging was the most widely used analysis method.

The neuroimaging studies in animal models showed that idiopathic RBD was attributed to selective lesions located in the pontine tegmentum, which was equivalent to the locus subcoeruleus in humans (Boeve et al., 2007). Furthermore, through VBM analysis, researchers demonstrated GM volume reduction in the anterior lobes of the right and left cerebellum, the tegmental portion of the pons, and the left parahippocampal gyrus (Hanyu et al., 2012) and increases of GM densities in both hippocampi (Scherfler et al., 2011) in patients with idiopathic RBD. In PD patients with RBD, Garcia-Lorenzo et al. (2013) showed reduced signal intensity in the coeruleus/subcoeruleus complex by neuromelanin-sensitive imaging but no evident changes in GM volume. Lim et al. (2016) analyzed the changes of GM volume at a whole-brain level, illustrating that RBD in PD may be related to decreased GM volume in the left posterior cingulate and hippocampus, but no structural abnormality was observed in the brainstem. Rahayel et al. (2019) applied VBM and deformation-based morphometry methods to analyze the regional difference in contraction or expansion between PD patients with and without RBD but still detected no significant results in the brainstem. In summary, although brainstem abnormality is found to be associated with the promotion of idiopathic RBD from experimental and clinical perspectives (Scherfler et al., 2011; Hanyu et al., 2012), there are no apparent volume changes of GM in the brainstem in PD patients with RBD compared with those without RBD.

Previous studies have demonstrated the strong association of reduced GM volume changes in the right STG with weakened spatial processing (Ellison et al., 2004; Gharabaghi et al., 2006; Shah-Basak et al., 2018), narcolepsy (Joo et al., 2009; Weng et al., 2015), impaired emotion processing to support social interactions (Muller et al., 2008; Pan et al., 2015; Van de Vliet et al., 2018; Zhang et al., 2018), violent behaviors (Zhang et al., 2019), and some psychiatric disorders (Moreira et al., 2017; Zhao et al., 2017; Wang et al., 2018). Consistent with our result, sleep disorders and nocturnal violence in PD patients with RBD can be partially explained by GM volume reduction in the right STG.

GM volume reduction in the right STG was associated with motor and non-motor manifestations, accounting for the RBD-related function impairment in PD patients. The presence of RBD in PD is associated with increased frequency of depression and falling (Romenets et al., 2012). Significant decrease in GM density was detected in the right superior temporal pole of PD patients with depression compared with that without depression (Feldmann et al., 2008). According to previous studies, STG plays a substantial role in the vestibular system and is associated with spatial information processing (Janzen et al., 2008). Consistently, Otomune et al. found that GM volume was significantly smaller in the right STG of frequent fallers than that of non-frequent fallers (Otomune et al., 2019). Besides, they found a significant linear correlation between fall frequency and GM reduction in the right STG (Otomune et al., 2019). Together, these findings supported an essential role of the right STG in the mental and postural control of PD patients with RBD. Psychosis is the most disabling non-motor complication in PD (Ffytche et al., 2017). STG is involved in processing visual and auditory information (Reale et al., 2007). Pacchetti et al. (2005) revealed that RBD increased the risk of symptomatic hallucinations and delusions in PD patients, showing the association of RBD with psychotic symptoms in PD. Additionally, there existed an inverse relationship between the severity of hallucinations and the right STG volume in early-onset schizophrenia (Matsumoto et al., 2001). Besides, a functional MRI study showed the association of reduced STG activity with auditory hallucinations (Orlov et al., 2018). Thus, our finding can partly help explain the neural substrate of association between RBD and PD psychosis. PD patients with RBD tend to develop a more rapid progression in cognition dysfunction (Fereshtehnejad et al., 2015). Reduced GM volume in the right STG is associated with poor ability to overcome misdirection (Tong et al., 2019), partly explaining its contribution to cognitive worsening in PD patients with RBD. In summary, RBD-related presentations in PD could be to some extent attributed to GM atrophy in the right STG, which can serve as a predictive tool for disease progression in PD.

The present study has some limitations that merit comment. First, polysomnography is mandatory for the diagnosis of RBD; however, the diagnosis of RBD in the research by Ford et al. was assessed by the Mayo Sleep Questionnaire, a well-validated tool for clinical screening (Ford et al., 2013). Second, the relatively small sample size of included studies limited the power of our meta-analysis using SDM-PSI methods. Third, VBM analysis is considered to be less sensitive in detecting regional abnormalities than cortical thickness analysis (Borghammer et al., 2010). Fourth, there exists heterogeneity of participants' characteristics between studies and various methodologies in VBM studies in terms of preprocessing protocols, smoothing kernels, and statistical thresholding methods.

## Conclusion

The result of our meta-analysis demonstrates that PD patient with RBD is associated with reduced GM volume of the right STG, suggesting a potential imaging biomarker for disease progression in PD. In the future, more functional neuroimaging and neurobiological studies are needed to provide further insights into how regional brain areas affect sleep disorders in PD patients.

## Author Contributions

CY wrote the manuscript. CY and JC performed the literature search and collected data. CY and XL performed the data analysis. XB and RW conceived of the ideas and reviewed the manuscript. All authors approved the manuscript for publication.

## Conflict of Interest

The authors declare that the research was conducted in the absence of any commercial or financial relationships that could be construed as a potential conflict of interest.
