# Supplementary material for: Gray Matter Alterations in Parkinson's Disease With Rapid Eye Movement Sleep Behavior Disorder: A Meta-Analysis of Voxel-Based Morphometry Studies
Source: Front Aging Neurosci. 2020 Aug 12;12:213. doi: 10.3389/fnagi.2020.00213 (PMC7434963; doi:10.3389/fnagi.2020.00213)
Supplement: Supplementary file 1 [file Table_1.DOCX]

Table S1. Quality assessment of five included studies in the present meta-analysis.

| Study | Subjects | | | | Methods for image acquisitions and analysis | | | | | | | Results and conclusions | | Total score |
| --- | --- | --- | --- | --- | --- | --- | --- | --- | --- | --- | --- | --- | --- | --- |
|  | Patients | Healthy comparisons | Variables | Sample size | Neuroanatomic measurements | > 1T | Slice thickness | No gap width | Clear imaging technique | Clear measurements | Inter-rater reliability | Statistical parameters | Consistent conclusions and limitations |  |
| Ford et al | 0.5 | 0 | 1 | 1 | 1 | 1 | 1 | 1 | 1 | 1 | 1 | 1 | 1 | 11.5 |
| Salsone et al | 1 | 1 | 1 | 1 | 1 | 1 | 1 | 1 | 1 | 1 | 1 | 1 | 1 | 13 |
| Kim et al | 1 | 1 | 1 | 1 | 1 | 1 | 1 | 1 | 1 | 1 | 1 | 1 | 1 | 13 |
| Lim et al | 1 | 1 | 1 | 1 | 1 | 1 | 1 | 0 | 1 | 1 | 1 | 1 | 1 | 12 |
| Rahayel et al | 1 | 1 | 1 | 1 | 1 | 1 | 1 | 1 | 1 | 1 | 1 | 1 | 1 | 13 |
